# IncRNAs transcriptomics elucidates the potential mechanism of Naoshuantong capsule in alleviating synaptic dysfunction in a murine model of cerebral ischemia/reperfusion injury

**DOI:** 10.3389/fphar.2026.1722930

**Published:** 2026-03-05

**Authors:** Ke Song, Hongrui Zhang, Haoqi Liu, Yuanyuan Li, Yikun Sun, Xinglu Dong, Chenxi Tao, Yannan He, Zhenhong Liu, Yonghong Gao, Ying Gao

**Affiliations:** 1 Key Laboratory of Chinese Internal Medicine of Ministry of Education and Beijing, Dongzhimen Hospital, Beijing University of Chinese Medicine, Beijing, China; 2 Institute for Brain Disorders, Beijing University of Chinese Medicine, Beijing, China; 3 Integrated Traditional and Western Medicine, The First Affiliated Hospital of Zhengzhou University, Zhengzhou, Henan, China; 4 Department of Neurology, Dongzhimen Hospital, Beijing University of Chinese Medicine, Beijing, China

**Keywords:** ischemic stroke, long noncoding RNA, Naoshuantong capsule, synaptic plasticity, transcriptome analysis

## Abstract

**Background:**

Naoshuantong capsule (NST), a Traditional Chinese Medicine formulation, is used for ischemic stroke treatment; however, its molecular mechanisms are unclear. This study aimed to investigate the mechanistic basis of NST using long noncoding RNA (lncRNA) and messenger RNA (mRNA) transcriptomics.

**Methods:**

The metabolites of NST were analyzed. Additionally, its systemically absorbed metabolites (in plasma) and brain-distributed metabolites were identified using ultrahigh-performance liquid chromatography–tandem mass spectrometry (UHPLC-MS/MS). The therapeutic effects of NST were evaluated in a mouse model of middle cerebral artery occlusion (MCAO) using neurological scoring, behavioral testing, cerebral blood flow, and brain tissue staining. LncRNA and mRNA expression profiles were analyzed using the Agilent Mouse competing endogenous RNA microarray, followed by gene ontology and Kyoto encyclopedia of genes and genomes enrichment analyses. Differentially expressed transcripts were validated using quantitative reverse transcription polymerase chain reaction (qRT-PCR).

**Results:**

UHPLC-MS/MS analysis detected 129 metabolites in NST; 33 metabolites in plasma; and 17 metabolites in brain tissue of rats administered with NST. NST treatment significantly reduced neurological deficit scores (Longa score), decreased beam-crossing latency, and increased forelimb grip strength in middle MCAO mice, indicating improved neurological function. Additionally, NST treatment enhanced cerebral blood flow recovery, ameliorated pathological damage, restored neuronal architecture, and increased Nissl-stained neuron density in peri-infarct brain tissue. NST also attenuated cellular apoptosis by upregulating Bcl-2 expression and downregulating Bax protein levels, exerting neuroprotective effects. Notably, NST treatment reversed 177 out of 5,378 differentially expressed IncRNAs and 52 out of 5,540 differentially expressed mRNAs that were dysregulated between the model and sham groups. These NST-modulated IncRNAs participate in key biological processes, including synaptic modulation, apoptosis regulation, and neuronal function. A synaptic plasticity-associated lncRNA-mRNA coexpression network was developed using NST-reversed transcripts. Validation using qRT-PCR confirmed the upregulation of NONMMUT050688.2 and NONMMUT044667.2, and the downregulation of NONMMUT092269.1 and NONMMUT101071.1, the downregulation of Nrn1, the upregulation of Grn, and the downward trend in Rasd2 expression in MCAO mice. All these alterations were reversed through NST treatment. *In vivo* experiments confirmed the efficacy of NST in ameliorating memory deficits, mitigating synaptic structural damage, and upregulating key synaptic protein expression (SYN and PSD95) in mice.

**Conclusion:**

NST may protect against cerebral ischemia/reperfusion injury by modulating lncRNA and mRNA expressions to enhance synaptic plasticity, thereby preserving neuronal structure and function.

## 1 Introduction

Globally, stroke is the second leading cause of mortality (GBD, 2019 Stroke Collaborators, 2021). Ischemic stroke (IS), which accounts for over 80% of all stroke cases, is characterized by high incidence, substantial disability, and high recurrence rates (Hu et al., 2017). The pathophysiological mechanism of IS is complex, encompassing damage to various signal pathways and multiple pathological processes, including excitotoxicity, free radical circulation, neuroinflammation, and reactive oxygen species generation (Sun et al., 2025; Zhao G. J. et al., 2025; Ahad et al., 2020). The current clinical treatment of IS, based on the pathophysiological characteristics of its onset, primarily focuses on ultra-early thrombolysis, neuroprotection in the acute stage, and repair of neurovascular structure and function in the recovery stage. The strict treatment time window constraints for intravenous thrombolysis significantly limit its application. Furthermore, some patients continue to exhibit neurological impairment and hemorrhagic transformation despite treatment (Liu et al., 2023; Shi et al., 2021). Clinical prognosis is mostly determined by the extent of ischemia-induced neuronal damage (Wang et al., 2023). Given that synaptic communication and adaptable circuit connections constitute the fundamental basis for sensation, voluntary movement, emotional regulation, and advanced cognition (Han and Wang, 2024; Wang et al., 2023), understanding and enhancing synaptic plasticity is crucial for facilitating neurological recovery after cerebral ischemia.

Long noncoding RNAs (lncRNAs) dynamically regulate key biological pathways, including cytogenesis, proliferative signaling, and developmental processes, with central nervous system-specific effects on neurogenesis, glial homeostasis, and synaptic functional plasticity (Li et al., 2025; Lin et al., 2025). lncRNAs are characterized as RNA molecules with over 200 nucleotides in length that lack protein-coding capacity (Mercer et al., 2009). Emerging evidence indicates that lncRNAs may significantly influence the onset of IS by regulating processes including cell apoptosis, inflammatory reaction, angiogenesis, neuroprotection, and tissue repair (Feng and Liu, 2025; He G. D. et al., 2025; Jiang et al., 2024). The role of lncRNAs in the underlying mechanisms and clinical progression of IS pathophysiology and patient outcomes has been extensively elucidated. Notably, these molecules demonstrate significant potential as valuable tools for enhancing early diagnostic accuracy, facilitating accurate disease subtyping, and enabling more reliable prognostic forecasts (Xu R. et al., 2025; Elhorany et al., 2024). Consequently, lncRNAs probably exert essential functions during the damaging and reparative stages of IS.

Traditional Chinese medicine (TCM) has a well-established theoretical framework for stroke treatment and offers unique advantages. Naoshuantong capsule (NST) comprises *Typhae* pollen, *Curcumae Radix*, *Gastrodiae Rhizoma, Radix Paeoniae Rubra,* and *Radix Rhapontici*. NST is a Category 3 New Drug developed in China, grounded in Academician Wang Yongyan’s “Toxin-Damaging Brain Collaterals” theory. NST markedly enhances neurological impairment, cognitive function, and self-care ability among patients with cerebral infarction and also enhances the efficacy of nerve injury repair, indicating substantial clinical benefits (Yang et al., 2023; Que et al., 2024). Furthermore, NST reduces infarct volume, repairs damaged brain tissue, and enhances coagulation, fibrinolysis, collateral microcirculation, and neural function at the lesion site in ischemic stroke animals. These effects are achieved by inhibiting inflammatory responses, exerting antioxidant effects, scavenging free radicals, and inhibiting the necrosis and apoptosis of ischemic cells (Liu et al., 2014). Our previous meta-analysis of NST in IS treatment demonstrated increased overall response rates, improved neurological function, elevated adiponectin levels, and reduced neurological impairments (Zhang et al., 2019). However, despite these promising findings, the precise molecular mechanisms, especially the role of lncRNA regulation, by which NST mediates its therapeutic efficacy against IS, remain unknown. This knowledge gap highlights the need for a comprehensive investigation into the transcriptomic landscape modulated by NST.

To address this gap and motivated by the quest for innovation, we utilized lncRNA microarray technology to delineate the comprehensive transcriptomic profiles (encompassing lncRNA and mRNA) in an ischemia-reperfusion mouse model subjected to NST intervention. This study provides the first comprehensive profiling of lncRNA and mRNA expression changes in response to NST treatment, representing a significant innovative aspect of our research. The biological functions and signaling pathways potentially associated with the markedly altered transcripts were examined using subsequent gene ontology (GO) and Kyoto encyclopedia of genes and genomes (KEGG) pathway analyses. We utilized quantitative reverse transcription polymerase chain reaction (qRT-PCR) to confirm the differential expression of representative lncRNAs and mRNAs. This integrated approach - combining unbiased RNA microarray, bioinformatics analysis, and experimental validation - was innovatively employed to identify novel potential mechanisms underlying the therapeutic effects of NST in IS. Therefore, this study aimed to identify novel therapeutic targets. Identifying such targets for NST is crucial, as it offers a systematic theoretical basis and scientific rationale to enhance its clinical application in IS management.

## 2 Materials and methods

### 2.1 Reagents and standards

Nissl Stain Kit was procured from Beijing Solarbio Science & Technology Co., Ltd. (Beijing, China; Catalog Number: G1432). Hematoxylin Staining Solution and Eosin Staining Solution were obtained from Beijing Zhongshan Jinqiao Biotechnology Co., Ltd. (Beijing, China; Catalog Numbers: ZLT-9610 and ZLT-9613, respectively). The DeadEnd™ Fluorometric TUNEL System was sourced from Promega Corporation (Madison, Wisconsin, United States; Catalog Number: G3250). RIPA lysis buffer was procured from Beijing Pulilai Gene Technology Co., Ltd. (Beijing, China; Catalog Number: C1053). The PSD95 rabbit monoclonal antibody was obtained from Cell Signaling Technology (Danvers, MA, United States; Catalog Number: 3409s). Anti-Synaptophysin (SYN) rabbit monoclonal antibody, anti-Bax rabbit monoclonal antibody, anti-Bcl-2 rabbit monoclonal antibody, and anti-beta Actin rabbit polyclonal antibody were obtained from Abcam (Cambridge, United Kingdom; Catalog Number: ab32127, ab32503, ab182858, and ab8227, respectively). Edaravone injection (National Drug Standard Approval Number: H20031342) was procured from Simcere Pharmaceutical Group Co., Ltd. (Nanjing, China). Reference standards (paeoniflorin, gastrodin, albiflorin, typhaneoside, isorhamnetin-3-O-neohesperidoside, hydroxyecdysone, and cryptochlorogenic acid) were obtained from Shanghai Yuanye Bio-Technology Co., Ltd., (Shanghai, China; Catalog Number: 23180-57-6, 62499-27-8, 39011-90-0, 104472-68-6, 55033-90-4, 5289-74-7, and 905-99-7, respectively).

### 2.2 Preparation of Naoshuantong capsule

Naoshuantong capsule (National Drug Standard Approval Number: Z20040093), obtained from Guangdong Huanan Pharmaceutical Co., Ltd. (production batch number: 210801), are composed of five botanical drugs: *Typha angustifolia L*. [Typhaceae; Typhae Pollen, TP], *Paeonia lactiflora Pall*. *[Paeoniaceae; Paeoniae Radix Rubra, PR], Curcuma longa L. [Zingiberaceae; Curcumae Radix, CR], Gastrodia elata Blume [Orchidaceae; Gastrodiae Rhizoma, GR], and Rhaponticum uniflorum (L.) DC. [Asteraceae; Rhapontici Radix, RR]*. All botanical drugs were authenticated to comply with the quality standards of the Chinese Pharmacopoeia 2020 edition (Volume I) through microscopic identification and thin-layer chromatography (TLC) by the Quality Inspection Department of Guangdong Zhongsheng Pharmaceutical Co., Ltd. The capsules were formulated with a defined ratio of ingredients: 33.33% TP, 23.78% PR, 19.10% CR, 9.55% GR, and 14.23% RR. The manufacturing procedure involved initial subjecting of PR to double reflux extraction with 70% ethanol (1 h each), followed by filtration, ethanol recovery, concentration, drying, pulverization, and mixing with calcium hydrogen phosphate to produce a dry extract powder. Simultaneously, CR was subjected to double reflux extraction using 80% ethanol (1 h each), with its filtered extract reserved. The resulting herb residue was subsequently combined with TP, GR, and RR for a double water decoction (1 h each). The combined decoction was filtered and concentrated to a paste with a relative density of 1.04–1.10 (measured at 40 °C). Ethanol was added to this paste to achieve a 70% alcohol concentration, and the mixture was refrigerated for 48 h. The supernatant was subsequently combined with the reserved CR ethanol extract, after which ethanol was recovered, and the blend was concentrated, dried, and pulverized. This powder was mixed with the previously prepared PR dry extract powder and an appropriate amount of calcium hydrogen phosphate. The mixture was granulated utilizing a hydroxypropyl methylcellulose ethanol solution, dried, and blended with talc, silicon dioxide, and magnesium stearate before being filled into capsules.

### 2.3 UHPLC-MS/MS and HPLC-ELSD/DAD analysis of metabolites in NST

For UHPLC-MS/MS analysis, a Thermo QE Plus liquid chromatography-tandem high-resolution mass spectrometer was utilized to identify the metabolites and those that permeate the blood and brain in the NST formulation. The Ion source voltage was set at 3.2 kV, and the auxiliary gas heater temperature was maintained at 350 °C. The sheath gas flow rate was sustained at 40 L/min. An ACQUITY UPLC HSS T3 column (2.1 × 100 mm, 1.8 μm) was selected for analysis. The mobile phase was composed of A (deionized water containing 0.1% formic acid) and B (acetonitrile containing 0.1% formic acid), utilizing gradient elution. The flow rate was set to 0.3 mL/min, and the column temperature was 35 °C. Data acquisition and analysis were conducted using Compound Discover data processing software.

The HPLC-ELSD/DAD analysis was performed on an Agilent 1260 Infinity II HPLC system equipped with a GRACE Prevail C18 column (5 μm, 4.6 × 250 mm) maintained at 35 °C. The mobile phase consisted of (A) acetonitrile with 0.1% formic acid and (B) 0.1% aqueous formic acid, using a gradient elution program at a flow rate of 1.0 mL/min. An evaporative light scattering detector (ELSD) was employed, with the evaporator and nebulizer temperatures both set at 65 °C and a gas flow rate of 1.6 SLM.

### 2.4 Animals group and treatment

Seven-week-old male C57BL/6J mice (weighing 25 ± 2 g; sourced from Beijing Vital River Laboratory Animal Technology Co., Ltd.) were maintained in specific pathogen-free facilities at Dongzhimen Hospital, Beijing University of Chinese Medicine. The living conditions were maintained at a temperature of 22 °C–24 °C with a 12-h light/dark cycle. The experimental procedures were approved by the Animal Care & Welfare Committee of Dongzhimen Hospital (Approval No. 21-25).

Mice were randomly allocated to four study groups: (1) sham group, (2) model group, (3) model + naoshuantong group (NST group, 0.468 g/kg), and (4) model + edaravone group (EDA group, 7.8 mg/kg).

On day 0, all animals in each group were anesthetized with pentobarbital sodium (56 mg/kg, i.p.). Following anesthesia, all groups except the sham group were subjected to middle cerebral artery occlusion (MCAO). For MCAO induction, the right MCA was occluded by inserting a silicon-coated monofilament through the intraluminal approach (Guangzhou Jialing Biotech). The filament was maintained at the occlusion site for 2 h to achieve ischemia, after which it was removed to initiate reperfusion. Sham mice underwent similar anesthesia and surgical procedures, except for the filament insertion. Cerebral blood flow (CBF) was continuously assessed during MCAO using Laser Speckle Contrast Imaging (LSCI; RFLSI III, RWD Life Science, China). Successful occlusion was defined as a decrease in CBF to <30% of the pre-ischemic baseline. Throughout surgery, body temperature was maintained at 37 °C with a heating pad. To ensure analgesia, local infiltration of 2% lidocaine at the incision site was performed prior to surgery.

Based on the clinical dosage for a 70 kg patient—3.6 g of NST capsules daily (three capsules of 0.4 g each, three times per day)—the equivalent dose for mice was calculated as 0.468 g/kg according to the body surface area-based conversion ratio between humans and experimental animals. At 2 h after reperfusion following MCAO, the NST group of mice was orally administered naoshuantong capsule (0.468 g/kg, via gastric gavage, prepared in 0.5% sodium carboxymethyl cellulose [Na-CMC]), while receiving an equal volume of saline via intraperitoneal injection. The edaravone group was treated with edaravone injection (7.8 mg/kg, via intraperitoneal injection) simultaneously with the same volume of 0.5% Na-CMC by gavage. The sham and model groups both received an equal volume of 0.5% Na-CMC via gavage and saline via intraperitoneal injection. All treatments were continued once daily until the day 7.

### 2.5 Neurological function assessment

#### 2.5.1 Evaluation of neurological scores

Neurological function was assessed on postoperative day 7 following MCAO induction utilizing the Longa scoring system (Longa et al., 1989), a 5-point scale where: 0 = normal function; 1–3 = progressive impairment; 4 = severe neurological deficits. (Note: Higher scores indicate greater deficit severity).

#### 2.5.2 Grip strength test

Grip strength, indicating neuromuscular function, was evaluated in experimental mice utilizing a grip strength tester (Bioseb, BIO-GS3, France), following the methodology described by Larcher et al. (2014). Each mouse was gently positioned on the grip plate, enabling the forelimbs to grasp the metal rod. The tail was subsequently steadily pulled backward, compelling the animal to apply maximum grip strength. The connected apparatus automatically recorded the peak grip strength. Three consecutive trials were performed per mouse, with mean values calculated for statistical analysis.

#### 2.5.3 Balance beam test

Mice were assessed for sensorimotor coordination and limb function using the balance beam test (Crabbe et al., 2003), with performance manually quantified using beam traversal time (Panlab, LE782, Barcelona, Spain).

### 2.6 Hematoxylin and eosin (H&E), Nissl, and TUNEL staining

The brains of mice in all groups were excised immediately on days 3 and 7 after reperfusion. H&E and Nissl staining were performed in strict accordance with the manufacturer’s instructions. Pathological sections were examined using a light microscope. TUNEL staining was performed strictly following the protocols of the DeadEnd™ Fluorometric TUNEL System. After TUNEL labeling, cells were counterstained using DAPI as a fluorescent tracer to visualize nuclei.

### 2.7 Novel object recognition (NOR) test

The NOR test was performed to evaluate cognitive function and consists of two distinct phases: training and testing (Ennaceur, 2010). The habituation period consisted of a 10-min unrestricted exploration session in an empty open-field arena for each mouse. In the training session, mice were given free access to explore two identical objects for 10 min. Subsequently, the testing phase was executed, during which one of the familiar objects (F) was substituted with a novel object (N). The mice were subsequently reintroduced to the arena for a 5-min session, during which their behavior was recorded. Recognition memory was assessed using a recognition index (RI), calculated for each animal as (N - F)/(N + F).

### 2.8 Transmission electron microscopy (TEM)

Electron microscopy was conducted as previously described (Zhang et al., 2015). The brain was immediately dissected and sectioned into small tissue blocks (approximately 2 mm^3^) for TEM processing. The brain tissue blocks preserved in the electron microscope fixative were washed, refixed, dehydrated, embedded, and polymerized into resin blocks, and subsequently sliced and examined under TEM (Hitachi H-7650, Tokyo, Japan). Synapse number, synaptic cleft width, postsynaptic density (PSD) thickness, and synaptic active zone length were measured using ImageJ software.

### 2.9 Microarray analysis and KEGG and GO analyses

Total RNA was extracted from cerebral cortices of mice in sham, model, and NST groups (n = 6/group) using TRIzol reagent (Invitrogen) according to the manufacturer’s instructions. Transcriptome profiling employed the Agilent Mouse Competing Endogenous RNA Microarray 2019 (4*180K, Design ID: 086242) across all 18 samples. Differentially expressed lncRNAs and mRNAs were identified using t-tests, with thresholds of |fold change| ≥ 2.0 and *P* ≤ 0.05. Differentially expressed lncRNAs were functionally annotated based on their predicted target mRNAs, utilizing current GO and KEGG databases. The significance level for enrichment was set at *P* < 0.05.

### 2.10 Quantitative reverse transcription polymerase chain reaction (qRT-PCR)

Quantitative polymerase chain reaction amplification was performed using a LightCycler® 480 II system (Roche, Switzerland) with a 10 μL reaction mixture containing 1 μL cDNA, 5 μL of 2 × PerfectStart™ Green qPCR SuperMix, 0.2 μL of each of the forward/reverse primers, and 3.6 μL nuclease-free water (Xie et al., 2023). The thermal profile comprised initial denaturation (94 °C, 30 s), followed by 45 cycles of 94 °C for 5 s and 60 °C for 30 s. The relative quantification of gene expression, normalized to GAPDH, was assessed using the 2^−ΔΔCT^ method. Primers (Table 1) were designed in-house based on lncRNA/mRNA sequences obtained from the NCBI databases and were synthesized by Tsingke Biotechnology Co., Ltd. (Beijing, China).

**TABLE 1:** qRT-PCR primers for IncRNAs or mRNAs.

Gene Symbol	Forward primer (5′–3′)	Reverse primer (5′-3′)
NONMMUT050688.2	TCCTCCGTGCCCATTGAA	CAAGAACACCAGTGACGTAA
NONMMUT092269.1	TTCCTGACCCACAAGG	CGA​GGA​GGT​GGT​ATG​CAT​GT
NONMMUT101071.1	GGTCATCAGCTCTGGCTA	GACTGAAGCCATGAGGCA
NONMMUT044667.2	GAT​GGC​CTC​AAC​CTG​GAC​TG	TGT​GGA​AGT​GAG​TAG​CAG​CC
Nrn1	ACG​TGA​CAG​GGT​TTT​TGC​TG	GGG​TAG​TTG​GCC​ATG​CTG​TC
Grn	AGG​CCT​CTT​GCT​GTG​AAG​AC	TCT​TAG​CAT​CAG​GGC​ACA​CG
Rasd2	CAG​TGG​GAA​CTG​CAC​ACT​CA	AGA​CAA​TGG​AGC​TCT​TGC​CC
mmu GAPDH	GCA​AGG​ACA​CTG​AGC​AAG​A	GGA​TGG​AAA​TTG​TGA​GGG​AG

### 2.11 Immunohistochemical analysis

The pathological sections were deparaffinized and rehydrated using an ethanol gradient and washed with phosphate-buffered saline (PBS). Subsequently, antigen retrieval was performed by subjecting the sections to microwave treatment for 10 min (Wang et al., 2024). After antigen retrieval, the sections underwent three sequential washes in PBS, each lasting 10 min. The samples were subsequently blocked and permeabilized with goat serum and incubated overnight at 4 °C with primary antibodies (anti-SYN at a 1:200 dilution; anti-PSD95 at a 1:400 dilution). Sections were washed thrice in PBS (10 min per wash), incubated with secondary antibody at room temperature for 30 min, and then washed again in PBS (3 × 10 min). Hematoxylin counterstaining (1 min) preceded microscopic examination.

### 2.12 Western blot

Total protein was extracted from the harvested brain tissues using RIPA lysis buffer. Aliquots (30 μg) underwent 10% sodium dodecyl sulfate–polyacrylamide gel electrophoresis separation and electrophoretic transfer to polyvinylidene fluoride membranes. After blocking in 5% non-fat milk solution, sequential antibody incubations were performed: initially with primary antibodies, followed by secondary antibody treatment (Zeng et al., 2024). The following primary antibodies were utilized for immunoblotting: PSD95 (1:3,000), synaptophysin (SYN) (1:50,000), Bax (1:1,000), Bcl-2 (1:2,000), and β-Actin (1:5,000). ImageJ software was utilized to determine the relative integrated density of the immunoblot bands.

### 2.13 Statistical analysis

Statistical analyses were conducted using the Statistical Product and Service Solutions software. Ordinal data are presented as median and interquartile range (IQR) and were analyzed using the Kruskal-Wallis test. Continuous data are expressed as the mean ± standard deviation (SD), and differences were assessed by one-way ANOVA followed by the Tukey-Kramer *post hoc* test for multiple comparisons. Significance thresholds were set at *P* < 0.05 and *P* < 0.01.

## 3 Results

### 3.1 Identification of metabolites in NST and its absorbed metabolites in blood and brain

**FIGURE 1:**
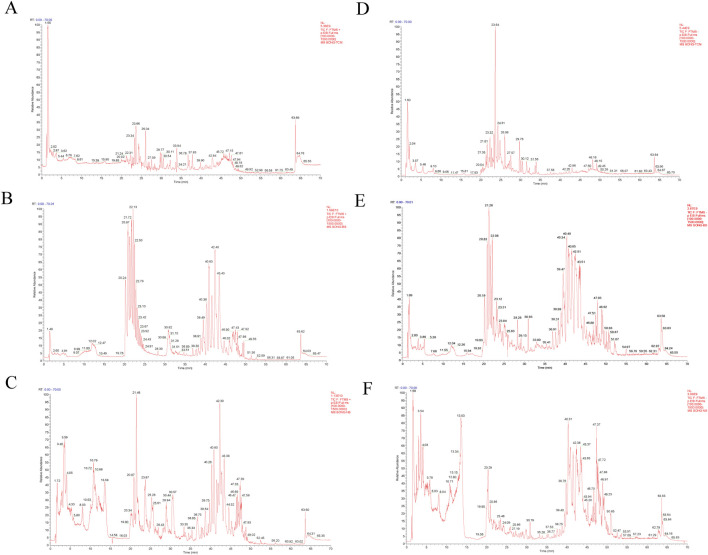
The total ion chromatogram (TIC) of NST by UHPLC-MS/MS analysis. **(A–C)** Positive ion mode. **(D–F)** Negative ion mode. **(A,D)** Total ion chromatogram of NST. **(B,E)** Total ion chromatogram of NST-containing plasma. **(C,F)** Total ion chromatogram of NST-containing brain.

The UHPLC-MS/MS analysis was employed to comprehensively characterize the metabolites of NST and to monitor its absorbed metabolites *in vivo*. We successfully identified 129 distinct metabolites within the NST. Following oral administration of NST, analysis of drug-containing plasma samples identified 33 metabolites originating from the formulation. Our analysis of NST-containing brain samples revealed that 17 metabolites derived from NST successfully penetrated the blood-brain barrier (BBB). Putative identification of chromatographic features was systematically performed by interrogating the online mzCloud™ and our in-house mzVault™ databases, specifically curated for TCM and natural products. Seven key metabolites—paeoniflorin, gastrodin, albiflorin, typhaneoside, isorhamnetin-3-O-neohesperidoside, hydroxyecdysone, and cryptochlorogenic acid were definitively identified by comparison with authentic reference standards analyzed under identical chromatographic and mass spectrometric conditions. Figure 1 depicts the representative total ion chromatograms for the NST, NST-containing plasma, and NST-containing brain. Supplementary Tables S1–S3 depict the detailed list of identified metabolites in NST, plasma, and brain tissue, respectively. In addition, we have performed two additional fingerprinting analyses—HPLC-ELSD (for non-UV absorbing compounds) and HPLC-DAD—which together with the UHPLC-MS/MS provide a comprehensive chemical profile meeting pharmacopeial standards for the characterization of polyherbal mixtures (Supplementary Figures S1–S5).

### 3.2 NST ameliorates neurological deficits and promotes CBF recovery in MCAO mice

**FIGURE 2:**
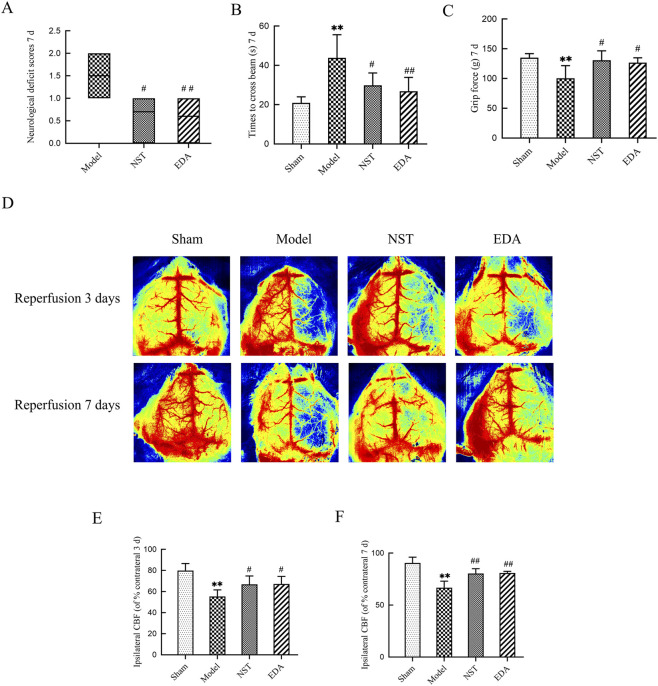
NST alleviated neurological deficits and promoted CBF recovery in MCAO mice. **(A)** Neurological deficit scores on day 7 post-MCAO (n = 10). **(B)** Balance beam crossing times on day 7 post-MCAO (n = 10). **(C)** Forelimb grip strength on day 7 post-MCAO (n = 10). **(D–F)** Representative images and quantitative analysis of CBF on days 3 and 7 post-MCAO (n = 10).

Herein, the neurological function was assessed in mice using the Longa score, the balance beam test, and the grip strength test. Furthermore, laser speckle contrast imaging was utilized to record the changes in CBF. Figures 2A–C depict that on day 7 post-operation, mice in the model group exhibited a significantly higher neurological deficit score than those in the sham group. However, those in the NST-treated group exhibited a significantly lower score than those in the model group (Figure 2A, *P* < 0.05). In the balance beam test, mice in the model group exhibited significantly longer duration to cross the beam than those in the sham controls (*P* < 0.01). Seven days of NST administration significantly reduced this crossing latency compared to the model group (Figure 2B, *P* < 0.05). Grip strength assessment revealed that forelimb grip strength among mice in the model group exhibited a significant decrease compared to that among mice in the sham group on day 7 post-ischemia-reperfusion (*P* < 0.01). NST treatment significantly increased forelimb grip strength compared to the model group (Figure 2C, *P* < 0.05). Figures 2D–F depict that at days 3 and 7 post-cerebral ischemia-reperfusion, the model group exhibited significantly reduced CBF compared to the sham group (*P* < 0.01). Administration of NST significantly improved CBF compared to the model group, with recovery observed on days 3 (*P* < 0.05) and 7 (*P <* 0.01). These data indicated that NST treatment facilitated the recovery of neurological deficits and CBF following MCAO.

### 3.3 NST ameliorates pathological damage, attenuates apoptosis, and exerts neuroprotection in MCAO mice

**FIGURE 3:**
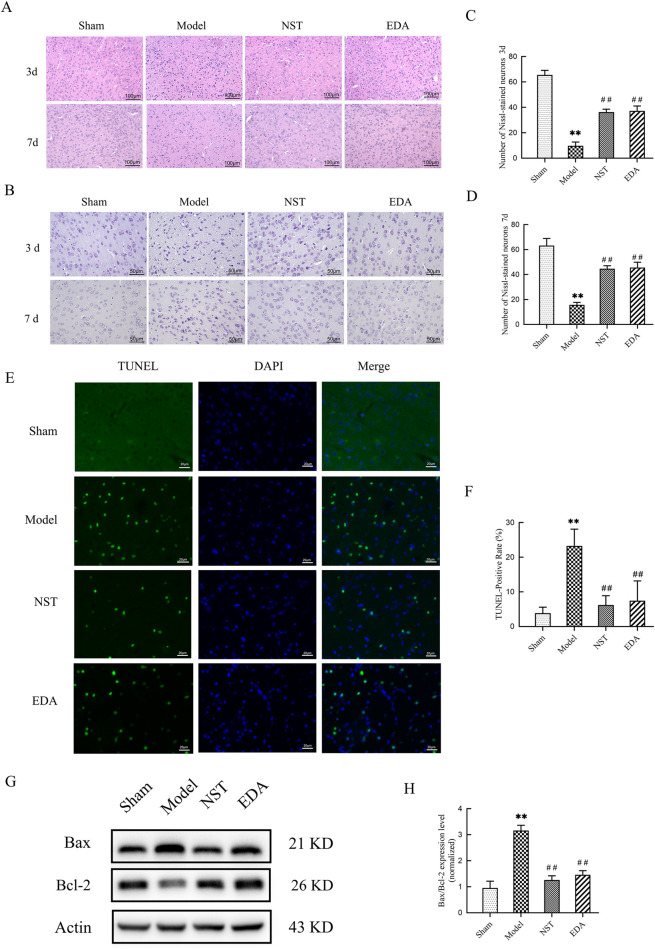
Neuroprotective effects of NST: amelioration of pathology and attenuation of apoptosis in MCAO mice. **(A)** Representative H&E-stained cortical sections illustrating histopathological changes. **(B)** Representative Nissl-stained cortical sections. **(C,D)** Quantitative analysis of Nissl-positive neurons in the cortex (n = 5). **P < 0.01 *versus* sham group; ^##^ *P* < 0.01 *versus* model group. **(E,F)** TUNEL-positive cells in the peri-infarct cortex were quantified (n = 6). **P < 0.01 *versus* sham group; ^##^ *P* < 0.01 *versus* model group. **(G,H)** The protein levels of the apoptosis-related proteins Bax and Bcl-2 were detected by Western blot in cortical lysates. (n = 4).**P < 0.01 *versus* sham group; ^##^ *P* < 0.01 *versus* model group.

Subsequently, we utilized H&E and Nissl staining to examine pathological alterations and neuronal damage in the peri-infarct region and TUNEL staining to evaluate apoptosis. Figure 3A displays that at days 3 and 7 post-ischemia-reperfusion, the peri-infarct brain tissue of the model group mice exhibited cellular morphological abnormalities, disorganized arrangement, and nuclear pyknosis with lysis. Post-NST treatment, these pathological features—including abnormal cellular morphology, cellular disarray, and nuclear pyknosis—were significantly ameliorated compared to the model group. Figures 3B–D indicate that Nissl staining revealed disorganized neuronal morphology and significantly reduced the number of Nissl-stained neurons in mice on days 3 and 7 post-ischemia-reperfusion compared to the sham group (*P* < 0.01). Notably, NST administration restored neuronal architecture and increased the number of Nissl-stained neurons compared to the model group at both time points (*P* < 0.01). TUNEL staining revealed extensive apoptotic activity in peri-infarct regions of mice in the model group, with TUNEL-positive cell counts significantly above those of the sham group (*P* < 0.01). After day 7 of NST treatment, this apoptosis was significantly reduced compared to the model group (*P* < 0.01, Figures 3E,F). Western blot analysis revealed a significantly increased Bax/Bcl-2 ratio in the model group compared to controls (*P* < 0.01). Treatment with NST significantly downregulated this pro-apoptotic ratio (*P* < 0.01, Figures 3G,H). These findings revealed that NST inhibits apoptosis following cerebral ischemic injury, probably through upregulation of Bcl-2 and downregulation of Bax protein expression, thus providing a neuroprotective effect. Collectively, our findings indicate that NST facilitates neuroprotection in MCAO mice by attenuating apoptosis.

### 3.4 Screening for NST-reversible IncRNAs and mRNAs in the cerebral cortex of MCAO mice

**FIGURE 4:**
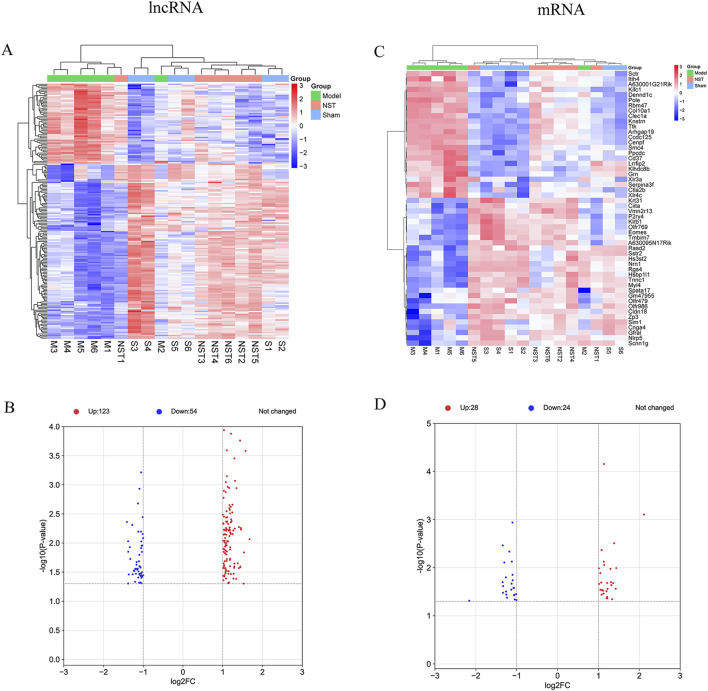
Screening of NST-reversible IncRNAs and mRNAs in the cerebral cortex of MCAO mice. **(A)** Hierarchical clustering heatmap of lncRNA expression profiles across groups. **(B)** Volcano plot illustrating NST-induced reversal of lncRNA expression. **(C)** Hierarchical clustering heatmap of mRNA expression profiles across groups. **(D)** Volcano plot demonstrating NST-induced reversal of mRNA expression.

Cerebral cortex tissues from model, NST, and sham groups were collected for high-throughput microarray analysis. Differentially expressed lncRNAs and mRNAs were identified using a fold change (≥2.0 or ≤0.5) and *P* values (<0.05) calculated by t-tests. Of the 5,378 differentially expressed lncRNAs identified between model and sham groups, 177 were reversed by NST treatment. Similarly, among the 5,540 differentially expressed mRNAs between these groups, 52 were reversed by NST. Heatmaps illustrating the expression values of lncRNAs and mRNAs across the model, sham, and NST groups revealed significant differences in expression patterns among the three groups (Figures 4A,C). Volcano plots (Figures 4B,D) further illustrate these reversals: among the lncRNAs and mRNAs upregulated in the model group compared to the sham group, 54 lncRNAs and 24 mRNAs were reverted to downregulation in the NST group. Among those downregulated in the model group, 123 lncRNAs and 28 mRNAs were reverted to upregulation in the NST group.

### 3.5 GO and KEGG enrichment analyses identify functional roles of NST-reversed IncRNAs in MCAO mice

**FIGURE 5:**
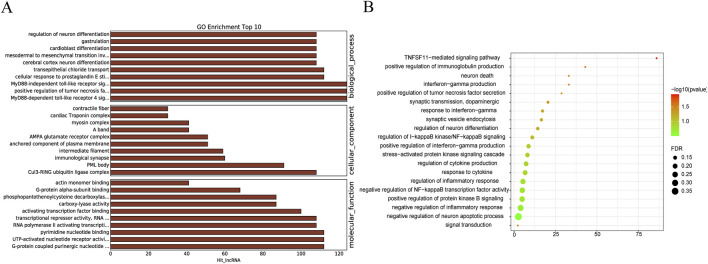
GO and KEGG enrichment analyses of differentially expressed IncRNAs reversed by NST. **(A)** Top 10 enriched Gene Ontology (GO) terms. **(B)** Enriched KEGG pathways.

To examine the molecular mechanism by which NST improves outcomes in IS, we selected lncRNAs whose notable expression changes were reversed by NST treatment. Subsequently, we analyzed the potential molecular functions of NST utilizing GO and KEGG pathway enrichment analyses. Our analysis revealed that the lncRNAs regulated by NST are involved in key biological processes. These include synaptic modulation (synaptic vesicle endocytosis, synaptic transmission, dopaminergic, immunological synapse), apoptosis (negative regulation of neuron apoptotic process, neuron death), and neuronal function regulation (regulation of neuron differentiation, cerebral cortex neuron differentiation, negative regulation of the inflammatory response) (Figures 5A,B).

### 3.6 Construction of an lncRNA-mRNA coexpression network for NST-reversed synaptic plasticity-associated transcripts and validation by qRT-PCR

**FIGURE 6:**
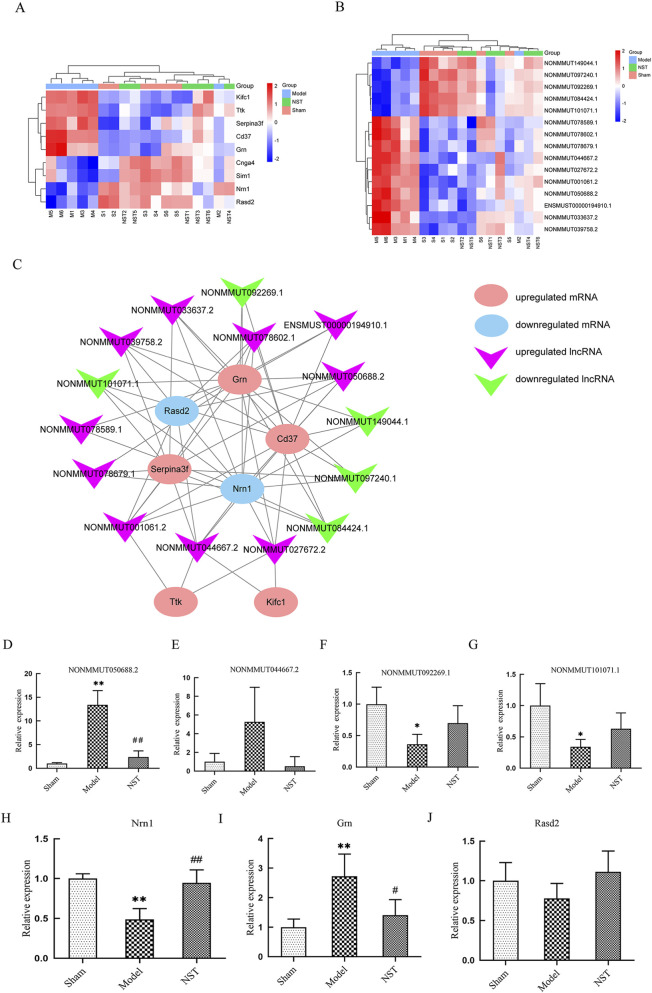
Coexpression network of IncRNAs and mRNAs in synaptic plasticity and its validation by qRT-PCR. **(A)** Heatmap of dysregulated mRNAs. **(B)** Heatmap of dysregulated IncRNAs. **(C)** Coexpression network of mRNAs and IncRNAs with expression significantly altered by NST treatment. Pink V-shapes denote upregulated IncRNAs; green V-shapes denote downregulated IncRNAs. Red ellipses represent upregulated mRNAs; blue ellipses represent downregulated mRNAs. **(D–J)** Validation of significantly altered transcripts by qRT-PCR.**P < 0.01 *versus* sham mice; ^#^ *P* < 0.05, ^##^ *P* < 0.01 *versus* model mice.

Considering the proven role of synaptic plasticity in post-stroke neural recovery, we analyzed synaptic plasticity-associated mRNAs whose expression was reversed by NST treatment in the mouse cerebral cortex, revealing nine differentially expressed mRNAs (Figure 6A). Subsequently, we calculated Pearson correlation coefficients between these mRNAs and all 177 analyzed lncRNAs that were reversed by NST treatment. An lncRNA-mRNA coexpression network was constructed based on significantly reversed mRNAs and lncRNAs. Figure 6C illustrates all lncRNA-mRNA pairs exhibiting a strong linear correlation (Pearson correlation coefficient >0.9 or < −0.9, *P* < 0.05). In this network, most lncRNAs seemed to be coexpressed with these mRNAs. Notably, seven mRNAs (Grn, Cd37, Nrn1, Rasd2, Ttk, Kifc1, and Serpina3f) are coexpressed with the top 15 differentially expressed lncRNAs (ten upregulated and five downregulated, Figure 6B), indicating that these lncRNAs might represent critical targets for NST in improving IS-related synaptic plasticity. To validate microarray findings, we conducted qRT-PCR analysis on randomly chosen differentially expressed lncRNAs and mRNAs. Additionally, qRT-PCR confirmed that two lncRNAs (NONMMUT050688.2 and NONMMUT044667.2) were upregulated, whereas NONMMUT092269.1 and NONMMUT101071.1 were significantly downregulated in MCAO mice (*P* < 0.05). Besides, Nrn1 was significantly downregulated (*P* < 0.01), Grn was significantly upregulated (*P* < 0.01), and Rasd2 exhibited a downward trend in MCAO mice, which was consistent with the microarray data (Figures 6D–J).

### 3.7 NST alleviates memory deficits, reduces synaptic structural damage, and increases the expression of SYN and PSD95 in MCAO mice

**FIGURE 7:**
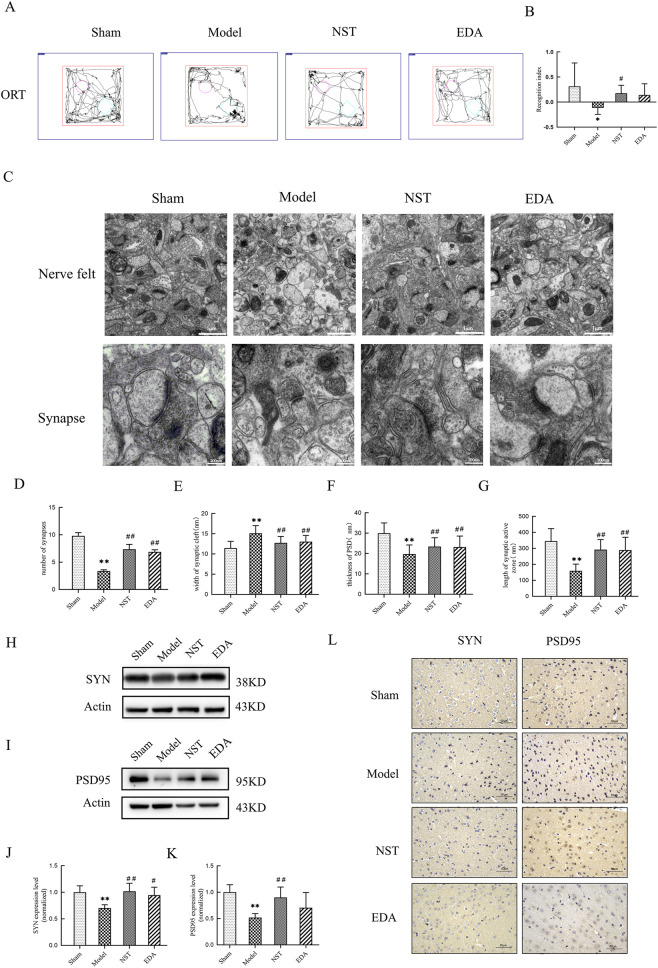
NST attenuates cognitive impairment and synaptic damage in MCAO mice. **(A)** Trajectory of ORT; **(B)** the recognition index, n = 8; **(C)** observation of nerve felt and synapse under electron microscope; **(D)** the number of synapses; **(E)** the width of synaptic cleft; **(F)** the thickness of PSD; **(G)** the length of synaptic active zone. **(H–K)** Western blot analysis was used to detect the protein expression levels of SYN and PSD95 in MCAO mice (n = 5). **(L)** Immunohistochemistry of SYN and PSD95. ORT: object recognition test.*P < 0.05, **P < 0.01 *versus* sham mice; ^#^ *P* < 0.05, ^##^ *P* < 0.01 *versus* model mice.

NOR was used to assess cognitive function in mice. Results demonstrated that on day 7 post-MCAO, NOR performance was significantly impaired among mice in the model group compared to those in the sham-operated group (*P* < 0.05). Following NST treatment, the NOR index in mice increased significantly (*P* < 0.05; Figures 7A,B). These findings revealed that NST can enhance learning and memory abilities in MCAO mice.

Electron microscopic analysis of synaptic microstructure revealed alterations in several synaptic connection parameters in MCAO mice compared to sham (Figures 7C–G). Specifically, these mice exhibited a reduced number of synapses, widened synaptic clefts, thinned PSD, and shortened synaptic active zones (all *P* < 0.01). NST treatment normalized these synaptic structural parameters. These results revealed that NST mitigates synaptic ultrastructural damage following IS.

Considering the established association between synaptic loss/dysfunction and cognitive impairment in patients with IS, we further evaluated the effect of NST on synaptic function by measuring the expression of synaptic-associated proteins Syn and PSD95. On day 7 post-MCAO, mice in the model group exhibited significantly lower expression of SYN and PSD95. However, NST treatment significantly increased SYN and PSD95 levels in the cerebral cortex of MCAO mice compared to the model group (Figures 7H–L).

## 4 Discussion

Globally, IS causes significant morbidity and is a leading cause of death (Lian et al., 2025; Ding et al., 2022). The pathophysiology of IS involves complex events, including oxidative stress, inflammation, autophagy, ferroptosis, and apoptosis (Cai et al., 2025; Liu Y. et al., 2025; Liu L. et al., 2025; Zhao H. et al., 2025). Emerging evidence demonstrates that lncRNAs, including ANRIL, MALAT1, and H19, play regulatory functions in these processes during IS (Ding et al., 2025; He P. et al., 2025; Pan et al., 2024). lncRNAs, characterized as transcripts lacking protein-coding potential, actively participate in essential biological processes through transcriptional regulation and post-transcriptional modulation of gene expression (Degirmenci et al., 2019). Numerous studies have demonstrated the therapeutic efficacy of NST in IS, including anti-inflammatory and anti-apoptosis effects, and reduction of cerebral infarction area (Zhang et al., 2019; Luo et al., 2020; Li et al., 2021). It is noteworthy that while prior studies, including work by our team (Xu T. et al., 2025) and others (Luo et al., 2020), have explored a range of doses (e.g., 0.468, 0.936, and up to 1.872 g/kg in Luo et al.), the present study focused specifically on the clinically equivalent dose of 0.468 g/kg. This dose was selected based on body surface area conversion and its established efficacy in ameliorating core pathological features of IS in models. We note that the administration of significantly higher doses (e.g., 1 g/kg/day and above, as occasionally reported in exploratory pharmacological literature) may increase the risk of non-specific effects or artifacts, and such doses require rigorous justification. This study reinforces the evidence for the protective effect of NST treatment in ameliorating IS in mice subjected to cerebral ischemia-reperfusion (MCAO). Our findings validated the successful MCAO model: mice exhibited significant neurological deficits, decreased CBF perfusion, and damage to the cerebral cortex and neurons. We conclusively proved that NST effectively ameliorates brain ischemia/reperfusion injury. However, the exact molecular mechanisms underlying NST’s beneficial effects in IS remain unknown. Therefore, to examine these mechanisms, we conducted microarray analysis to identify differentially expressed genes in the cerebral cortex of MCAO mice following NST treatment.

Analysis of gene expression profiles revealed 5,378 lncRNAs and 5,540 mRNAs differentially expressed in MCAO mice. Additionally, we identified 177 lncRNAs and 52 mRNAs whose expression levels were significantly reversed after NST treatment, demonstrating a potent regulatory influence of NST on cortical gene expression. This finding offers a genetic basis for understanding NST’s pharmaceutical mechanism in mitigating IS. GO and KEGG enrichment analyses revealed that these NST-regulated genes are predominantly associated with key pathophysiological processes of IS, including the following: synaptic modulation, apoptosis, and neuronal function regulation. These pathways signify critical pathological mechanisms targeted by NST to ameliorate cerebral ischemia reperfusion injury. Synaptic plasticity, a key molecular basis for neural function recovery post-ischemia, facilitates neural network reconstruction through the formation and stabilization of new synapses (Li et al., 2019). This is significantly essential given the prevalence of post-stroke cognitive impairment (affecting 20%–80% of patients) (Sun et al., 2014) and its occurrence in over one-third of patients with transient ischemic attack (van Rooij et al., 2014). A major mechanism underlying this cognitive impairment is the loss of synaptic proteins, including SYN and PSD95 (Sinclair et al., 2015). Our findings strongly indicate that enhancing synaptic plasticity is an essential mechanism underpinning NST’s neuroprotective effect against cerebral ischemic injury. Moreover, qRT-PCR validation of selected lncRNAs implicated in IS pathogenesis confirmed that these genes represent potential key targets for NST’s protective action.

From the lncRNAs associated with NST-mediated amelioration of IS, we selected four candidates for qRT-PCR validation: NONMMUT050688.2 and NONMMUT044667.2, which were upregulated in the MCAO model group and downregulated after NST treatment, and NONMMUT101071.1 and NONMMUT092269.1, which were downregulated in the model group and upregulated after NST treatment; qRT-PCR quantification successfully recapitulated the differential expression observed in the sequencing data for all four lncRNAs. Neuritin (Nrn1) is a neurotrophic factor mostly expressed in the nervous system, essential for nerve development, synaptic formation, and plasticity. *Nrn1* knockout mice exhibit impaired synaptic formation and learning deficits (Fujino et al., 2011), whereas *Nrn1* overexpression in transgenic mice enhances the expression of neurofilament protein-200, SYN, and GAP-43, and facilitates recovery of spatial learning and memory following ischemia-reperfusion injury (Wan et al., 2020). In this experimental stroke model, we demonstrated that immediate NST administration post-MCAO significantly ameliorated cerebral injury by suppressing cortical neuronal apoptosis, downregulating Bax expression, and upregulating Bcl-2, SYN, and PSD95 protein levels. This molecular modulation preserved synaptic ultrastructure integrity and accelerated functional recovery from MCAO-induced behavioral deficits, collectively establishing a mechanistic basis for NST-mediated enhancement of learning and memory capabilities following IS.

This study demonstrated that NST treatment markedly reverses the transcriptomic alterations induced by cerebral ischemia-reperfusion, including extensive dysregulation of lncRNAs. These findings indicate NST’s global impact on gene expression profiles and its specific targeting of lncRNA networks. Pathway enrichment analysis of significantly regulated genes implicates synaptic plasticity as the central mechanism underlying NST’s therapeutic efficacy against ischemic injury. Experimentally, immediate post-reperfusion NST administration enhanced synaptic plasticity, suppressed neuronal apoptosis, and improved functional recovery in MCAO mice. We identified synaptic plasticity-associated mRNAs co-regulated with numerous lncRNAs using coexpression network analysis. Our data substantiate a mechanistic model wherein NST facilitates synaptogenesis through lncRNA-mediated regulation of target mRNAs, revealing a promising therapeutic strategy for IS.

In this study, edaravone was employed as a positive control drug. Although edaravone is a single chemical entity, in contrast to the multi-component nature of NST, its selection was justified based on its established clinical efficacy in acute ischemic stroke and its well-documented neuroprotective properties, including the preservation of synaptic plasticity (Otani et al., 2005). This comparison provides a clinically relevant benchmark to evaluate the therapeutic potential of the complex formulation NST.

This study has several limitations. First, although mass spectrometry analysis of NST and subsequent *in vitro* and *in vivo* experiments aimed to identify neuroactive metabolites that enter the blood-brain barrier, comprehensive pharmacodynamic profiling of these metabolites requires additional studies. Second, for transcriptomic analysis, we chose microarray technology due to its quantitative accuracy in detecting known transcripts, which was crucial for our research objectives. While this approach reliably identifies differentially expressed genes within a well-annotated framework, it is limited in discovering novel transcripts or splicing variants. This limitation can be addressed in future studies using RNA-seq, which would complement our findings by identifying novel lncRNAs and expanding our understanding of the transcriptomic landscape.

Although lncRNAs may exhibit limited sequence conservation across species, their biological functions are primarily mediated through highly conserved cellular pathways rather than strict nucleotide sequence identity. Thus, functional conservation rather than sequence conservation is exactly the key determinant of translational relevance. Because processes such as neuroinflammation, synaptic plasticity regulation and neuronal apoptosis are fundamentally preserved between rodents and humans, lncRNAs identified in mice MCAO models can provide meaningful insights into pathological mechanisms relevant to human ischemic stroke. In our study, several differentially expressed lncRNAs were predicted to regulate downstream targets with established roles in human ischemic stroke, such as granulin (GRN) and serine protease inhibitor A3 (SERPINA3). GRN acts as a pro-inflammatory mediator produced via proteolytic cleavage of progranulin (PGRN) (Horinokita et al., 2019). Similarly, SERPINA3 is a novel inflammatory biomarker implicated in neurological disorders, including ischemic stroke (Hu et al., 2024). The convergence of these differentially expressed lncRNAs on gene pathways already validated in human patients further strengthens the clinical relevance of our findings. The observed co-expression of lncRNAs with key mRNAs (e.g., Nrn1, Grn, Serpina3f) involved in synaptic function and apoptosis suggests potential regulatory interactions. LncRNAs are known to regulate gene expression at both transcriptional (e.g., by modulating chromatin architecture) and post-transcriptional levels (e.g., by acting as competing endogenous RNAs (ceRNAs) to influence mRNA stability) (Bao et al., 2018). While the present microarray study provides a comprehensive overview and identifies candidate lncRNA-mRNA pairs, the precise mechanistic links—whether through direct transcriptional regulation, ceRNA networks, or other modes—require validation through targeted loss-of-function and gain-of-function experiments in future studies.

## 5 Conclusion and future perspectives

This study systematically demonstrated the multi-level mechanisms through which NST ameliorates cerebral ischemia-reperfusion injury. Our findings indicate that NST significantly reduces neurological deficits, facilitates CBF recovery, and alleviates neuronal pathological damage and apoptosis in MCAO mice, while also exerting neuroprotective effects by modulating lncRNAs and mRNA expression profiles in the cerebral cortex. Notably, NST specifically reverses the aberrant expression of lncRNAs (NONMMUT050688.2, NONMMUT044667.2, NONMMUT101071.1, and NONMMUT092269.1) and their coexpressed mRNAs (Nrn1 and Grn), which are closely linked to synaptic plasticity, apoptosis, and neuronal function. The molecular changes correlate with phenotypic improvements induced by NST, including enhanced expression of synaptic proteins (SYN and PSD95), improved synaptic ultrastructure, and greater learning and memory capabilities. This study is the first to reveal a novel mechanism from the perspective of lncRNA-mRNA coexpression networks, indicating that NST may facilitate post-stroke neurological recovery by modulating lncRNAs, thereby influencing target genes associated with synaptic plasticity.

Given that our findings are based on animal models, further clinical validation is necessary to assess the translational potential of these lncRNA targets. Moving forward, a key focus of our research will be conducting dynamic studies. These will involve time-series analyses to track the expression kinetics of key lncRNAs and their potential target mRNAs (such as Nrn1) following NST treatment.

## Data Availability

The original contributions presented in the study are publicly available. This data can be found here: https://doi.org/10.6084/m9.figshare.31302553. Further inquiries can be directed to the corresponding author(s).
