# Supplementary material for: IncRNAs transcriptomics elucidates the potential mechanism of Naoshuantong capsule in alleviating synaptic dysfunction in a murine model of cerebral ischemia/reperfusion injury
Source: Front Pharmacol. 2026 Mar 5;17:1722930. doi: 10.3389/fphar.2026.1722930 (PMC12999579; doi:10.3389/fphar.2026.1722930)
Supplement: Supplementary file 1 [file Supplementaryfile1.docx]

Supplementary Material

## 1 Supplementary Tables

Table S1 Characterization of the main metabolites in NST using UHPLC-MS/MS

| **Compound** | **Formula** | **Calc. MW** | **RT [min]** | **Area（serum）** |
| --- | --- | --- | --- | --- |
| Paeoniflorin* | C23 H28 O11 | 480.16255 | 23.762 | 23510101101 |
| Ethyl gallate | C9 H10 O5 | 198.05256 | 24.39 | 10278404343 |
| Citric acid | C6 H8 O7 | 192.02708 | 4.266 | 8954597660 |
| Parishin E | C19 H24 O13 | 460.12124 | 21.797 | 6164690089 |
| 1,2,3,4,6-Pentagalloylglucose | C41 H32 O26 | 940.11701 | 24.997 | 5950558354 |
| Albiflorin* | C23 H28 O11 | 480.16247 | 26.043 | 5660385083 |
| Benzoylpaeoniflorin | C30 H32 O12 | 584.18856 | 29.618 | 5279641270 |
| Cryptochlorogenic acid* | C16 H18 O9 | 354.0945 | 22.448 | 4709112667 |
| Gallic acid | C7 H6 O5 | 170.02136 | 8.599 | 3819901516 |
| Sucrose | C12 H22 O11 | 342.11593 | 2.239 | 3735134766 |
| Parishin C | C32 H40 O19 | 728.21567 | 22.858 | 2257657023 |
| (+)-Catechin hydrate | C15 H14 O6 | 290.07865 | 22.499 | 2206146467 |
| Naringenin | C15 H12 O5 | 272.06806 | 30.381 | 1965503150 |
| Ferulaldehyde | C10 H10 O3 | 178.06283 | 23.76 | 1874739014 |
| Paeonolide | C20 H28 O12 | 460.15782 | 22.889 | 1816192156 |
| Cantharidin | C10 H12 O4 | 196.07332 | 26.046 | 1750633253 |
| Ellagic acid | C14 H6 O8 | 302.00581 | 24.615 | 1663437409 |
| Oxypaeoniflorin | C23 H28 O12 | 496.15772 | 22.288 | 1596195066 |
| Isorhamnetin-3-O-nehesperidine* | C28 H32 O16 | 624.16806 | 24.488 | 1556012771 |
| 2-Pyrrolidinecarboxylic acid | C5 H9 N O2 | 115.06353 | 2.232 | 1447407162 |
| Hydroxyecdysone* | C27 H44 O7 | 480.30778 | 24.427 | 1391342606 |
| Gastrodin* | C13 H18 O7 | 286.10627 | 16.708 | 1334398363 |
| Paeonol | C9 H10 O3 | 166.06284 | 22.89 | 1322875571 |
| Pyrogallol | C6 H6 O3 | 126.03151 | 8.594 | 1293942633 |

Table S1: Continued

| **Compound** | **Formula** | **Calc. MW** | **RT [min]** | **Area（serum）** |
| --- | --- | --- | --- | --- |
| Typhaneoside* | C34 H42 O20 | 770.22573 | 23.841 | 1152776221 |
| Quinic acid | C7 H12 O6 | 192.06323 | 2.217 | 1146745874 |
| 1,3-Dicaffeoylquinic acid | C25 H24 O12 | 516.12617 | 23.31 | 1038250384 |
| Isorhamnetin | C16 H12 O7 | 316.05766 | 24.489 | 681058428.2 |
| α-Linolenic acid | C18 H30 O2 | 278.22423 | 47.501 | 617678297 |
| Cinnamaldehyde | C9 H8 O | 132.05736 | 26.047 | 572456738.9 |
| Procyanidin B1 | C30 H26 O12 | 578.14197 | 22.27 | 570575957 |
| Caffeic acid | C9 H8 O4 | 180.04215 | 21.539 | 480260935.7 |
| Kaempferol 3-glucorhamnoside | C27 H30 O15 | 612.16857 | 24.44 | 432391387 |
| Uridine | C9 H12 N2 O6 | 244.07029 | 6.47 | 431806652.4 |
| Arglabin | C15 H18 O3 | 246.12595 | 32.734 | 430159327.6 |
| Azelaic acid | C9 H16 O4 | 188.10511 | 26.203 | 430117917.4 |
| Parishin A | C45 H56 O25 | 996.30969 | 23.887 | 377623885.8 |
| Dehydrocostus lactone | C15 H18 O2 | 230.13053 | 33.952 | 364549967.2 |
| Ursolic acid | C30 H48 O3 | 456.35991 | 48.337 | 363754328.1 |
| Narcissoside | C28 H32 O16 | 624.16832 | 25.114 | 340622090 |
| 18 β-Glycyrrhetintic Acid | C30 H46 O4 | 470.33898 | 44.212 | 312989362.4 |
| Atractyloside A | C21 H36 O10 | 494.23617 | 27.001 | 298909347.1 |
| Atractylenolide I | C15 H18 O2 | 230.13042 | 41.734 | 287032335.8 |
| Baicalin | C21 H18 O11 | 446.08432 | 26.914 | 279893729.7 |
| (-)-Catechin gallate | C22 H18 O10 | 442.08948 | 24.73 | 253672883.6 |
| Nicotinamide | C6 H6 N2 O | 122.04876 | 4.187 | 252436876.3 |
| Kaempferol | C15 H10 O6 | 286.04729 | 24.428 | 247595663.8 |
| Manninotriose | C18 H32 O16 | 504.16905 | 3.22 | 226549418.2 |
| Nicotinic acid | C6 H5 N O2 | 123.03207 | 3.357 | 214973424 |
| Isoguanosine | C10 H13 N5 O5 | 283.09145 | 13.869 | 198141608.1 |
| Artemisinic acid | C15 H22 O2 | 234.16171 | 42.342 | 186616655.6 |
| Curcumenol | C15 H22 O2 | 234.16228 | 24.332 | 186212189 |
| Salicylic acid | C7 H6 O3 | 138.03195 | 26.664 | 185954055.6 |
| Mannitol | C6 H14 O6 | 182.07911 | 2.127 | 182978716 |
| Spiculisporic acid | C17 H28 O6 | 328.18853 | 38.922 | 172084561.2 |
| 3,5-Dicaffeoylquinic acid | C25 H24 O12 | 516.12624 | 25.388 | 171082907 |

Table S1: Continued

| **Compound** | **Formula** | **Calc. MW** | **RT [min]** | **Area（serum）** |
| --- | --- | --- | --- | --- |
| Cytosine | C4 H5 N3 O | 111.04386 | 3.578 | 170214100.8 |
| Parthenolide | C15 H20 O3 | 248.14151 | 33.92 | 167438066.5 |
| Grosvenorine | C33 H40 O19 | 740.21571 | 23.832 | 165832530 |
| Rutin | C27 H30 O16 | 610.15262 | 23.902 | 165324020.6 |
| α-Cyperone | C15 H22 O | 218.16686 | 46.862 | 163169182.9 |
| (-)-Epicatechin gallate | C22 H18 O10 | 442.08946 | 24.482 | 155231239.2 |
| Wogonoside | C22 H20 O11 | 460.10039 | 28.875 | 140969745.2 |
| 4-Hydroxybenzoic acid | C7 H6 O3 | 138.03165 | 22.074 | 136684160.5 |
| Isochlorogenic acid B | C25 H24 O12 | 516.12605 | 25.074 | 130392955.9 |
| Kaempferol-3-O-rutinoside | C27 H30 O15 | 594.15795 | 24.427 | 129370661.5 |
| Rhaponticin | C21 H24 O9 | 420.14171 | 25.801 | 118075328.9 |
| Germacrone | C15 H22 O | 218.16685 | 45.045 | 111158415.4 |
| Epicatechin | C15 H14 O6 | 290.07869 | 21.762 | 109281484.5 |
| Methyl gallate | C8 H8 O5 | 184.03682 | 22.475 | 98733254.34 |
| Isochlorogenic acid C | C25 H24 O12 | 516.12615 | 25.841 | 93441753.07 |
| Stachyose | C24 H42 O21 | 666.22204 | 5.35 | 92898525.9 |
| Phloridzin | C21 H24 O10 | 436.13636 | 26.276 | 89347160.02 |
| Astragalin | C21 H20 O11 | 448.10002 | 24.428 | 86726267.53 |
| Corilagin | C27 H22 O18 | 634.08003 | 22.324 | 85877769.04 |
| Cytidine | C9 H13 N3 O5 | 243.08688 | 3.573 | 78651681.45 |
| Pedunculoside | C36 H58 O10 | 696.40779 | 30.417 | 70463273.22 |
| Naringenin chalcone | C15 H12 O5 | 272.06798 | 24.45 | 67214766.41 |
| Deoxyandrographolide | C20 H30 O4 | 312.22963 | 42.933 | 66651166.5 |
| Atractylenolide II | C15 H20 O2 | 232.14668 | 26.757 | 64635009.85 |
| Protocatechuic acid | C7 H6 O4 | 154.02689 | 23.123 | 61505931.67 |
| Quercetin | C15 H10 O7 | 302.04228 | 23.902 | 61178394.06 |
| Shikimic acid | C7 H10 O5 | 174.05232 | 22.544 | 59815970.57 |
| Atractylenolide III | C15 H20 O3 | 248.14167 | 36.776 | 55986189.33 |
| Alnustone | C19 H18 O | 262.13545 | 44.715 | 55227578.43 |
| Oroxylin A-7-O-β-D-glucuronide | C22 H20 O11 | 460.10017 | 28.278 | 53110074.56 |
| Calcium pantothenate | C9 H17 N O5 | 219.11167 | 20.976 | 52020664.27 |

Table S1: Continued

| **Compound** | **Formula** | **Calc. MW** | **RT [min]** | **Area（serum）** |
| --- | --- | --- | --- | --- |
| Wogonin | C16 H12 O5 | 284.06821 | 34.989 | 51182177.87 |
| L-Tryptophan | C11 H12 N2 O2 | 204.09063 | 21.53 | 48635361.79 |
| Morin | C15 H10 O7 | 302.04228 | 23.384 | 44186245.72 |
| Glabrolide | C30 H44 O4 | 468.32366 | 40.039 | 43835662.43 |
| Cimifugin | C16 H18 O6 | 306.11065 | 24.917 | 43506032.18 |
| Dictamnine | C12 H9 N O2 | 199.06375 | 32.777 | 42809382.92 |
| Lupenone | C30 H48 O | 424.36995 | 47.558 | 41297336.36 |
| Decursinol angelate | C19 H20 O5 | 328.13063 | 40.594 | 37558438.29 |
| Taxifolin | C15 H12 O7 | 304.05768 | 25.225 | 36632485.1 |
| Columbianadin | C19 H20 O5 | 328.13063 | 41.54 | 35665732.08 |
| (+)-Magnoflorine | C20 H23 N O4 | 341.16249 | 23.319 | 30945864.78 |
| Raffinose | C18 H32 O16 | 550.17427 | 3.91 | 29642155.58 |
| Phloretin | C15 H14 O5 | 274.08398 | 26.264 | 29473786.11 |
| 7-Methoxycoumarin | C10 H8 O3 | 194.05775 | 47.36 | 27949775.97 |
| Ferulic acid | C10 H10 O4 | 194.05804 | 25.031 | 27589602.85 |
| Emodin | C15 H10 O5 | 270.05247 | 38.939 | 25743027.04 |
| Isorhapontigenin | C15 H14 O4 | 258.08885 | 25.32 | 25615337.52 |
| Dehydrodiisoeugenol | C20 H22 O4 | 326.15123 | 29.411 | 25410686.02 |
| Pinocembrin | C15 H12 O4 | 256.0733 | 35.576 | 24569489.52 |
| Baicalein | C15 H10 O5 | 270.05195 | 26.911 | 22249770.53 |
| Ethyl caffeate | C11 H12 O4 | 208.07327 | 29.28 | 21934094.2 |
| Vanillin | C8 H8 O3 | 152.04783 | 26.76 | 20816596.51 |
| Rhapontigenin | C15 H14 O4 | 226.0627 | 25.802 | 20480103.92 |
| Eriodictyol | C15 H12 O6 | 288.06304 | 28.302 | 19811742.91 |
| 5-O-Methylvisammioside | C22 H28 O10 | 452.16796 | 25.151 | 19372513.16 |
| 2-Adamantanone | C10 H14 O | 150.10436 | 25.883 | 18908702.16 |
| Oroxylin A | C16 H12 O5 | 284.06821 | 35.769 | 18869593.82 |
| Curculigoside | C22 H26 O11 | 466.14705 | 23.587 | 17598188.77 |
| Curcumin | C21 H20 O6 | 368.12558 | 37.474 | 15087348.92 |
| Fumaric acid | C4 H4 O4 | 116.01145 | 2.308 | 14659669.17 |
| Fraxinellone | C14 H16 O3 | 232.1103 | 38.417 | 13170205.19 |
| α-Boswellic acid | C30 H48 O3 | 456.36005 | 36.085 | 12125868.08 |

Table S1: Continued

| **Compound** | **Formula** | **Calc. MW** | **RT [min]** | **Area（serum）** |
| --- | --- | --- | --- | --- |
| Luteolin | C15 H10 O6 | 286.04824 | 28.538 | 11657965.36 |
| Apigenin | C15 H10 O5 | 270.05269 | 30.489 | 10118480.35 |
| Hesperetin | C16 H14 O6 | 302.07869 | 30.713 | 9773026.534 |
| β-Asarone | C12 H16 O3 | 208.1103 | 36.303 | 8225566.858 |
| Dehydrotrametenolic acid | C30 H46 O3 | 454.34416 | 40.949 | 6330352.918 |
| Chrysin | C15 H10 O4 | 254.05763 | 35.148 | 5412275.636 |
| Demethoxycurcumin | C20 H18 O5 | 338.1151 | 36.938 | 4935179.239 |
| Roburic acid | C30 H48 O2 | 440.36731 | 51.332 | 4519426.354 |
| Linolenic acid ethyl ester | C20 H34 O2 | 306.25744 | 49.065 | 2380556.002 |
| Bryodulcosigenin | C30 H50 O4 | 496.35524 | 52.851 | 1974865.438 |

Abbreviations: MW: Molecular weight, RT: Retention time.

*Metabolites confirmed by comparing with a reference standard

Table S2 Characterization of the main metabolites of NST into serum with UHPLC-MS/MS

| **Compound** | **Formula** | **Calc. MW** | **RT [min]** | **Area（serum）** |
| --- | --- | --- | --- | --- |
| Salicylic acid | C7 H6 O3 | 138.03195 | 26.664 | 2189091743 |
| Paeoniflorin* | C23 H28 O11 | 480.16255 | 23.762 | 2014801795 |
| Gastrodin* | C13 H18 O7 | 286.10627 | 16.708 | 862709511.1 |
| Parishin E | C19 H24 O13 | 460.12124 | 21.797 | 537022400.2 |
| Protocatechuic acid | C7 H6 O4 | 154.02689 | 23.123 | 335683851.5 |
| Paeonolide | C20 H28 O12 | 460.15782 | 22.889 | 257114540.1 |
| Paeonol | C9 H10 O3 | 166.06284 | 22.89 | 198810163 |
| Ferulaldehyde | C10 H10 O3 | 178.06283 | 23.76 | 167010066 |
| Oxypaeoniflorin | C23 H28 O12 | 496.15772 | 22.288 | 158077617.9 |
| Spiculisporic acid | C17 H28 O6 | 328.18853 | 38.922 | 74400962.02 |
| Sucrose | C12 H22 O11 | 342.11593 | 2.239 | 62225065.19 |
| Albiflorin* | C23 H28 O11 | 480.16247 | 26.043 | 61862813.53 |
| 4-Hydroxybenzoic acid | C7 H6 O3 | 138.03165 | 22.074 | 59577949.3 |
| Vanillin | C8 H8 O3 | 152.04783 | 26.76 | 53785580.39 |
| Curcumenol | C15 H22 O2 | 234.16228 | 24.332 | 52016427.42 |
| Parishin C | C32 H40 O19 | 728.21567 | 22.858 | 47282673.47 |
| Atractylenolide III | C15 H20 O3 | 248.14167 | 36.776 | 43749996.9 |
| Typhaneoside* | C34 H42 O20 | 770.22573 | 23.841 | 29624730.34 |
| Cimifugin | C16 H18 O6 | 306.11065 | 24.917 | 29465519.04 |
| Isorhamnetin-3-O-nehesperidine* | C28 H32 O16 | 624.16806 | 24.488 | 27038736.66 |
| Cantharidin | C10 H12 O4 | 196.07332 | 26.046 | 25535303.18 |
| Atractyloside A | C21 H36 O10 | 494.23617 | 27.001 | 24081573.94 |
| Arglabin | C15 H18 O3 | 246.12595 | 32.734 | 23589426.9 |
| Ethyl gallate | C9 H10 O5 | 198.05256 | 24.39 | 23118979.51 |
| Wogonoside | C22 H20 O11 | 460.10039 | 28.875 | 20384148.56 |
| Kaempferol 3-glucorhamnoside | C27 H30 O15 | 612.16857 | 24.44 | 15054234.73 |
| Cinnamaldehyde | C9 H8 O | 132.05736 | 26.047 | 10283769.27 |
| Manninotriose | C18 H32 O16 | 504.16905 | 3.22 | 10131019.61 |
| Naringenin | C15 H12 O5 | 272.06806 | 30.381 | 9175534.678 |
| Stachyose | C24 H42 O21 | 666.22204 | 5.35 | 4190574.683 |

Table S2: Continued

| **Compound** | **Formula** | **Calc. MW** | **RT [min]** | **Area（serum）** |
| --- | --- | --- | --- | --- |
| Baicalin | C21 H18 O11 | 446.08432 | 26.914 | 3550106.187 |
| Oroxylin A-7-O-β-D-glucuronide | C22 H20 O11 | 460.10017 | 28.278 | 3336331.472 |
| Roburic acid | C30 H48 O2 | 440.36731 | 51.332 | 1191497.483 |

Abbreviations: MW: Molecular weight, RT: Retention time.

*Metabolites confirmed by comparing with a reference standard

Table S3 Characterization of the main metabolites of NST into brain with UHPLC-MS/MS

| **Compound** | **Formula** | **Calc. MW** | **RT [min]** | **Area（brain）** |
| --- | --- | --- | --- | --- |
| Paeoniflorin* | C23 H28 O11 | 480.16255 | 23.762 | 687268631 |
| Gastrodin* | C13 H18 O7 | 286.10627 | 16.708 | 413616266 |
| Ethyl gallate | C9 H10 O5 | 198.05256 | 24.39 | 285163419.3 |
| Salicylic acid | C7 H6 O3 | 138.03195 | 26.664 | 249970006.2 |
| Arglabin | C15 H18 O3 | 246.12595 | 32.734 | 210744519.9 |
| Cryptochlorogenic acid* | C16 H18 O9 | 354.0945 | 22.448 | 71639381.2 |
| Albiflorin* | C23 H28 O11 | 480.16247 | 26.043 | 53175991.93 |
| Parishin E | C19 H24 O13 | 460.12124 | 21.797 | 40249579.15 |
| Curcumenol | C15 H22 O2 | 234.16228 | 24.332 | 38219748.05 |
| Atractylenolide II | C15 H20 O2 | 232.14668 | 26.757 | 37303151.95 |
| Atractylenolide III | C15 H20 O3 | 248.14167 | 36.776 | 36517429.37 |
| Dictamnine | C12 H9 N O2 | 199.06375 | 32.777 | 32513014.76 |
| Naringenin | C15 H12 O5 | 272.06806 | 30.381 | 30407619.79 |
| Parthenolide | C15 H20 O3 | 248.14151 | 33.92 | 28980918.65 |
| Dehydrocostus lactone | C15 H18 O2 | 230.13053 | 33.952 | 26704406.62 |
| Parishin C | C32 H40 O19 | 728.21567 | 22.858 | 11893337.24 |
| Spiculisporic acid | C17 H28 O6 | 328.18853 | 38.922 | 4323473.103 |

Abbreviations: MW: Molecular weight, RT: Retention time.

*Metabolites confirmed by comparing with a reference standard

## 2 Supplementary Figures

**
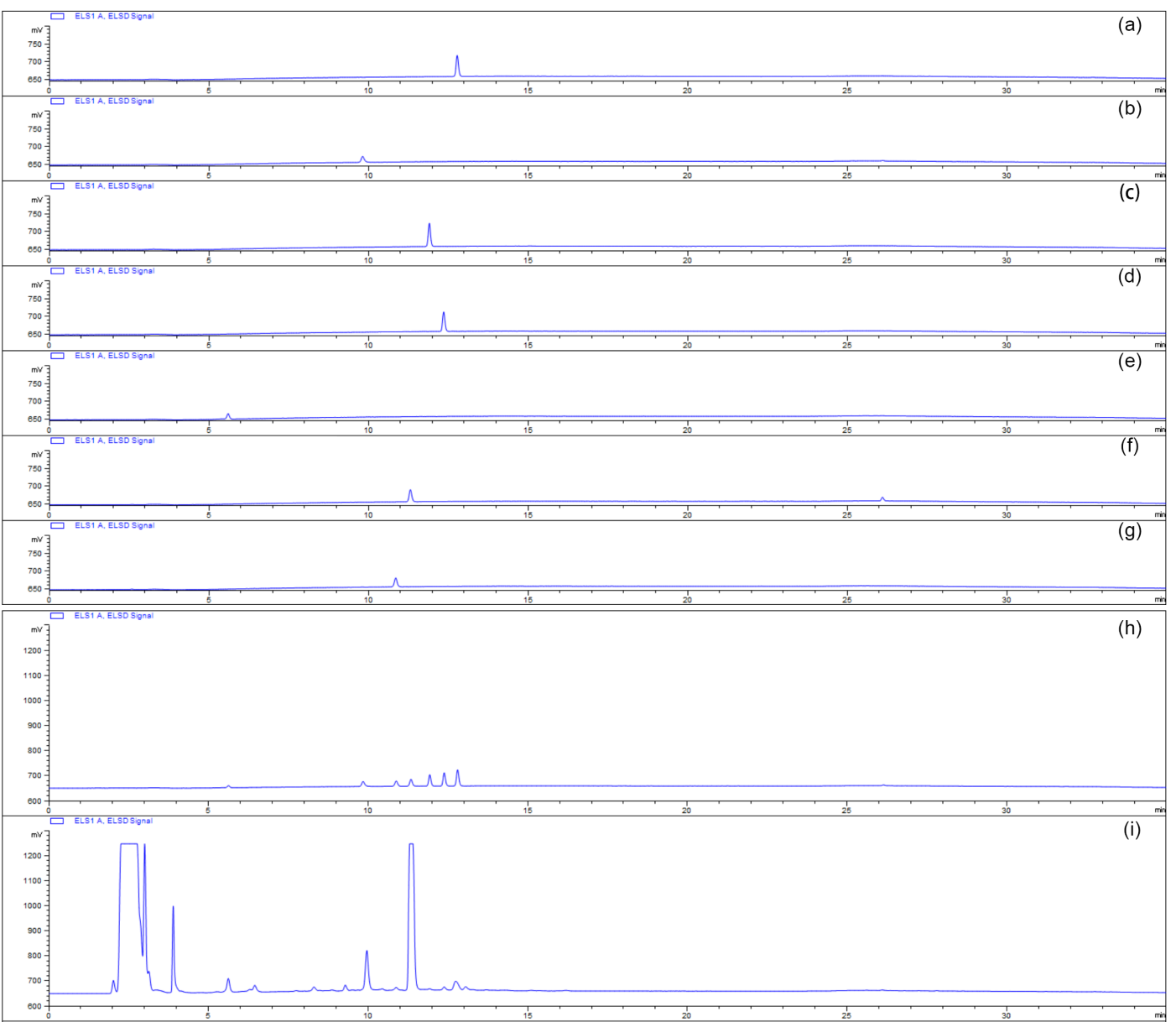
**

**Supplementary Figure S1.** HPLC-ELSD chromatograms of NST. (a) standard solution: isorhamnetin-3-O-neohesperidoside. (b) standard solution: cryptochlorogenic acid. (c) standard solution: typhaneoside. (d) standard solution: hydroxyecdysone. (e) standard solution: gastrodin. (f) standard solution: paeoniflorin. (g) standard solution: albiflorin. (h) mixed standard solution. (i) NST.

**
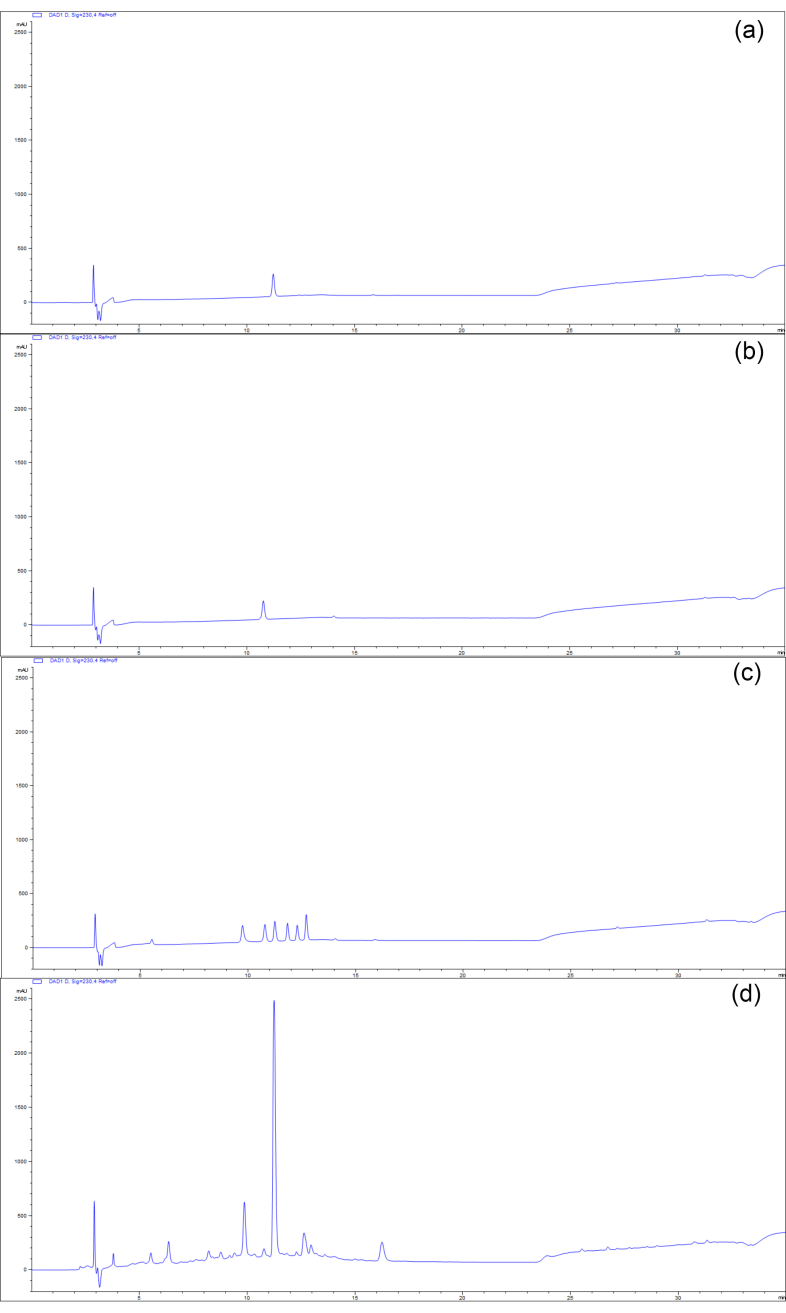
**

**Supplementary Figure S2.** HPLC-DAD chromatograms at 230 nm of NST. (a) standard solution: paeoniflorin. (b) standard solution: albiflorin. (c). mixed standard solution. (d). NST.

**
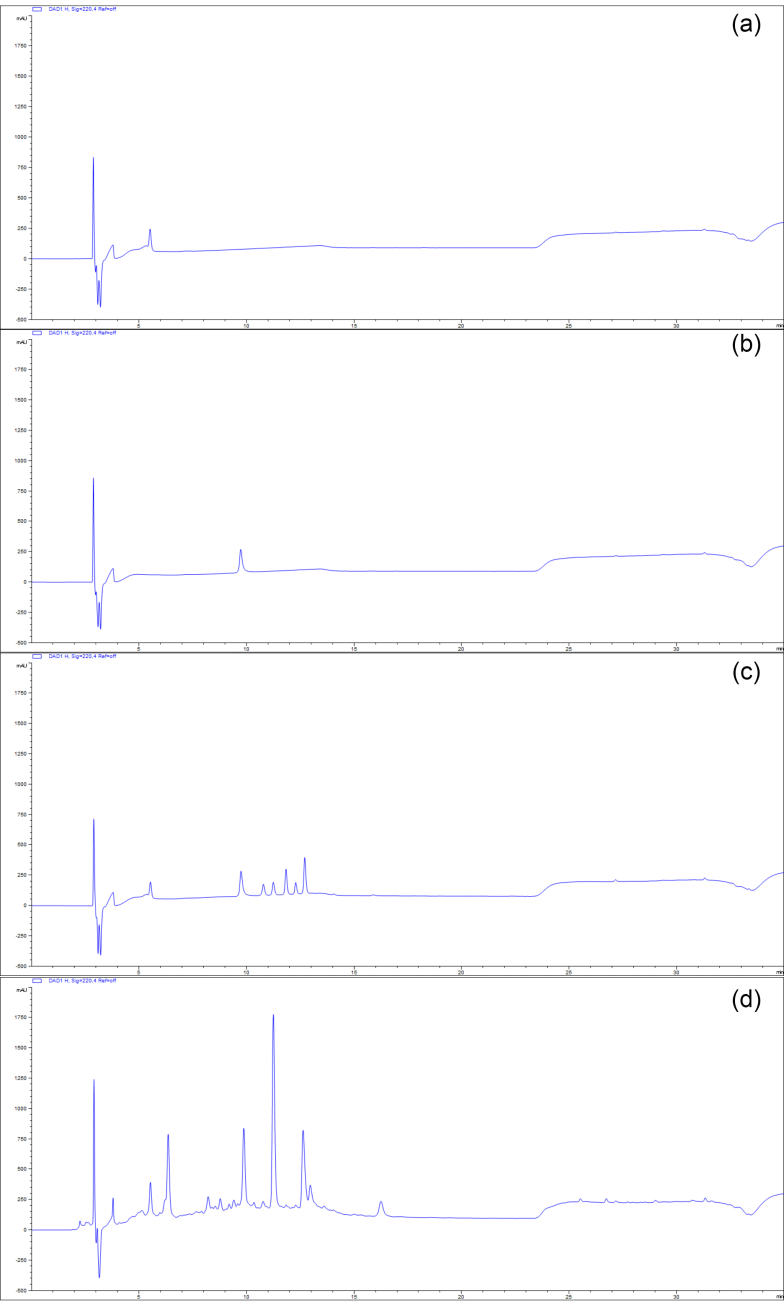
**

**Supplementary Figure S3.** HPLC-DAD chromatograms at 220 nm of NST. (a) standard solution: gastrodin. (b). standard solution: cryptochlorogenic acid. (c). mixed standard solution. (d). NST.


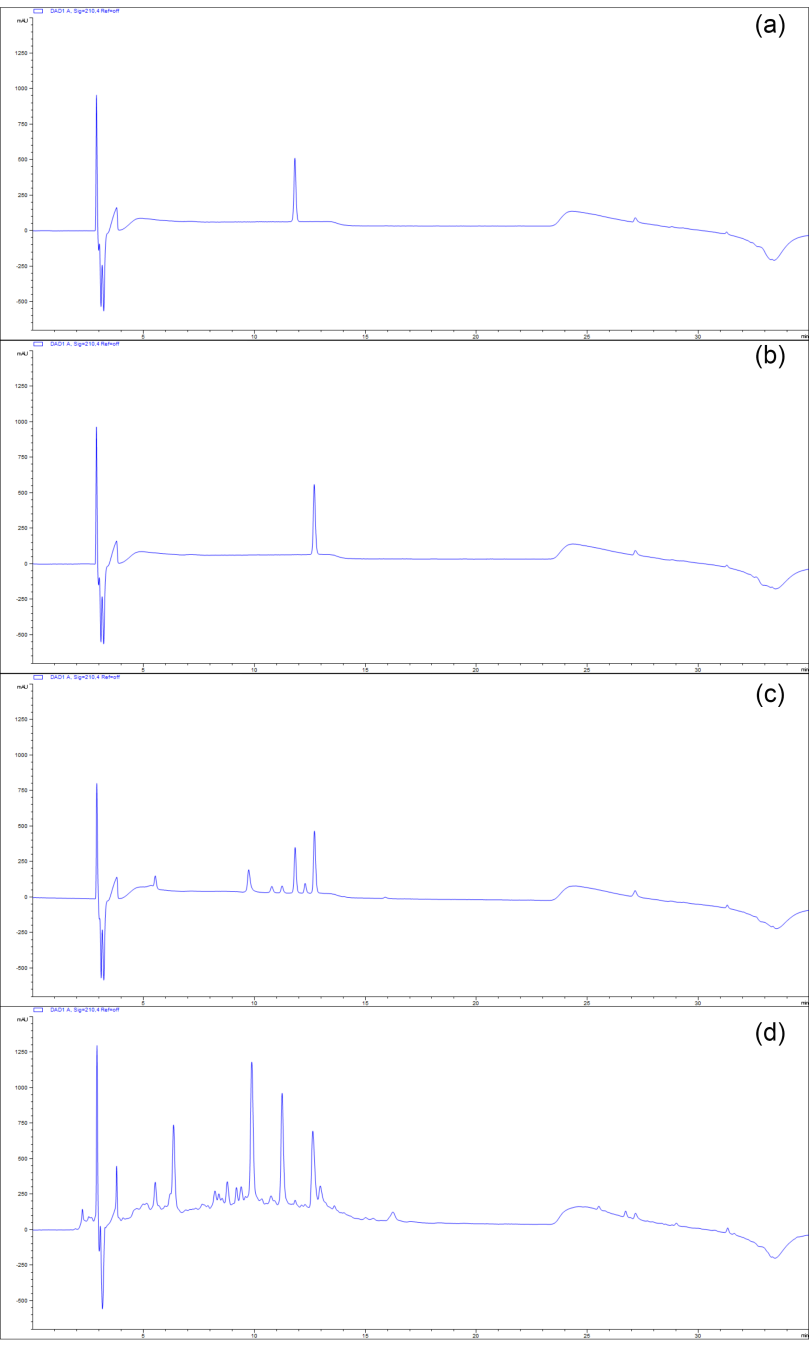


**Supplementary Figure S4.** HPLC-DAD chromatograms at 210 nm of NST. (a). standard solution: typhaneoside. (b). standard solution: isorhamnetin-3-O-neohesperidoside. (c). mixed standard solution. (d). NST.

**
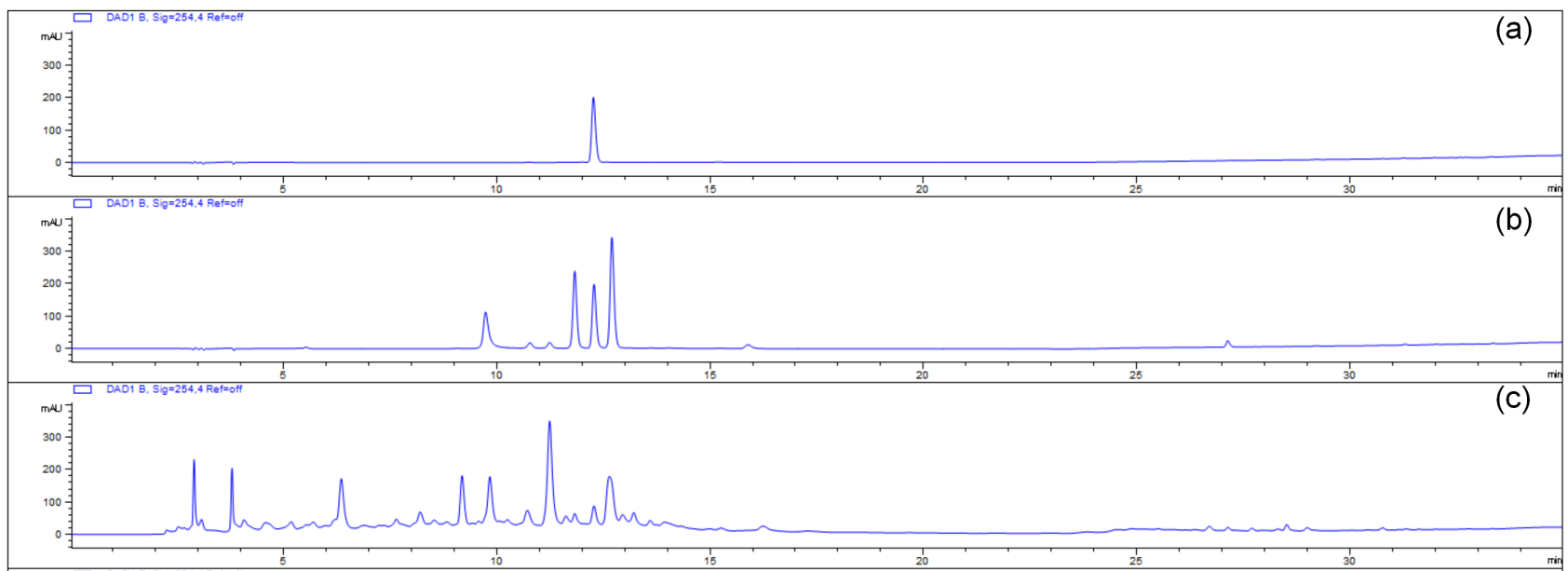
**

**Supplementary Figure S5.** HPLC-DAD chromatograms at 254 nm of NST. (a). standard solution: hydroxyecdysone. (b). mixed standard solution. (c). NST.
